# Macropinocytosis in phagocytes: regulation of MHC class-II-restricted antigen presentation in dendritic cells

**DOI:** 10.3389/fphys.2015.00001

**Published:** 2015-01-30

**Authors:** Zhenzhen Liu, Paul A. Roche

**Affiliations:** Experimental Immunology Branch, National Cancer Institute, National Institutes of HealthBethesda, MD, USA

**Keywords:** dendritic cell, macropinocytosis, endocytosis, MHC class II, antigen presentation

## Abstract

Dendritic cells (DCs) are outstanding antigen presenting cells (APCs) due to their robust ability to internalize extracellular antigens using endocytic processes such as receptor-mediated endocytosis, phagocytosis, and macropinocytosis. Macropinocytosis mediates the non-specific uptake of soluble antigens and occurs in DCs constitutively. Macropinocytosis plays a key role in DC-mediated antigen presentation to T cells against pathogens and the efficiency of macropinocytosis in antigen capture is regulated during the process of DC maturation. Here, we review the methods to study macropinocytosis, describe our current knowledge of the regulatory mechanisms of antigen uptake via macropinocytosis and the intracellular trafficking route followed by macropinocytosed antigens, and discuss the significance of macropinocytosis for DC function.

## Introduction

Dendritic cells (DCs) are the most accomplished of professional antigen presenting cells (APCs) and are responsible for initiating T cell responses against pathogens (Villadangos and Schnorrer, 2007). DCs are present throughout the body and function by sampling their microenvironment to “sense” pathogens. DCs express peptide fragments of degraded foreign antigens on their surface bound to MHC glycoproteins, molecules that function as “display platforms” presenting peptide-fragments for recognition by antigen-specific T cells (Blum et al., 2013). Peptide-MHC class I complexes (pMHC-I) are recognized by cytotoxic CD8^+^ T cells whereas peptide-MHC class II complexes (pMHC-II) are recognized by helper CD4^+^ T cells. Besides stimulating distinct classes of T cells, it is generally true that MHC-I and MHC-II differ in the sites of intracellular antigenic peptide generation. MHC-I-destined peptides are generated by cytosolic proteolysis of endogenous proteins and foreign (often viral) antigens, and these peptides are translocated into the endoplasmic reticulum for binding to nascent MHC-I. In specialized DC subsets, however, these peptides can be translocated directly into phagosomes for binding to MHC-I in a process termed cross-presentation (Joffre et al., 2012). Cross-presentation can also result from direct endo/lysosomal proteolysis of internalized antigens (Shen et al., 2004; Kreer et al., 2011), however this review will not address the endocytic mechanisms responsible for cross-presentation as this has been reviewed recently (Schuette and Burgdorf, 2014). By marked contrast with MHC-I, MHC-II-destined peptides are often generated by endo/lysosomal proteolysis of internalized pathogens. DCs generally recognize and capture foreign invaders in non-lymphoid organs. Upon pathogen capture, DCs concurrently mature and migrate to secondary lymphoid organs (such as lymph nodes) where their surface pMHC-I and pMHC-II present antigenic peptides to T cells to initiate antigen-specific adaptive immune responses.

**Figure 1:**
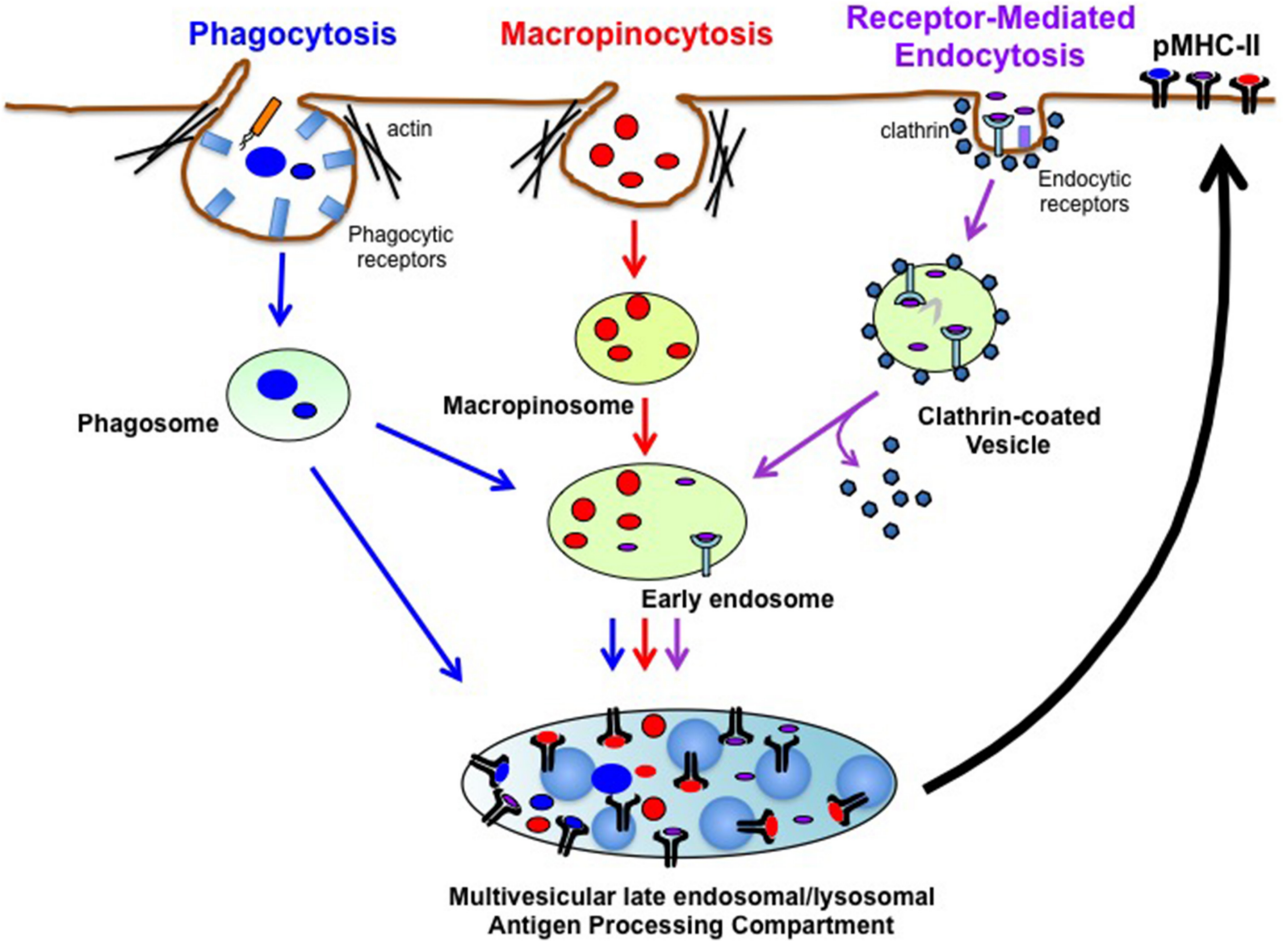
**Pathways of Exogenous Antigen Uptake in DCs**. DCs internalize extracellular antigens using three main endocytic pathways. Phagocytosis is an endocytic process in which opsonized particles bind to specific receptors on the DC surface and enter cells in membrane-derived phagosomes. Phagocytosed antigens (Blue) can fuse with MHC-II^+^ lysosomes to generate phagolysosomes (not shown) or can be directly targeted into late endosomal/lysosomal antigen processing compartments to generate pMHC-II complexes. Macropinocytosis mediates non-specific uptake of soluble antigens into the cell via macropinosomes. Antigens in macropinosomes (Red) are transferred into early endosomes that eventually fuse with multivesicular late endosomal/lysosomal antigen processing compartments. Macropinocytosed antigens are degraded and loaded on MHC-II in these compartments. Receptor-mediated endocytosis is a process in which small soluble antigens bind to specific receptors on the DC surface that internalize in clathrin-coated vesicles or in clathrin-uncoated vesicles (not shown). Following uncoating of clathrin, antigens in clathrin-coated vesicles (Purple) are delivered to early endosomes and eventually to antigen processing compartments for proteolytic degradation and pMHC-II formation. After formation in antigen processing compartments, pMHC-II complexes traffic to plasma membrane to initiate antigen-specific adaptive immune responses.

One feature that makes DCs particularly effective stimulators of CD4^+^ T cell responses is their considerable capacity to acquire exogenous antigens/pathogens by a variety of different endocytic processes (Figure 1). Receptor-mediated endocytosis requires antigen (usually small soluble molecules) binding to a variety of receptors on DCs (most notably lectins, Fc receptors, and complement receptors) that are internalized in clathrin-coated and non-clathrin-coated vesicles. Phagocytosis is a form of endocytosis that mediates the internalization of a wide-variety of relatively-large insoluble particulate antigens including necrotic/apoptotic cells and opsonized pathogenic organisms/viruses (Stuart and Ezekowitz, 2005). Like receptor-mediated endocytosis, phagocytosis is usually initiated by antigen binding to DC surface “receptors,” however, DCs can also internalize large particles (such as antigen-coated latex beads) by non-specific phagocytosis. Lastly, macropinocytosis (Lim and Gleeson, 2011), or “cell drinking,” is the non-specific uptake of soluble molecules, nutrients and antigens. By contrast, phagocytosis and receptor-mediated endocytosis mediate specific uptake of extracellular materials via a number of distinct surface receptors (Burgdorf and Kurts, 2008). Macropinocytosis is an actin-dependent process initiated from plasma membrane ruffles that give rise to large endocytic vesicles called macropinosomes, which are distinct from other forms of endocytic vesicles. Unlike small (0.1 μm) clathrin-coated vesicles, macropinosomes have no apparent coat structure and are heterogeneous in size ranging from 0.2 to 5 μm in diameter (Hewlett et al., 1994; Swanson and Watts, 1995). Macropinocytosis occurs constitutively in DCs and DCs generated *in vitro* from mouse bone marrow (BMDC) can internalize approximately 2 fL/cell/min (Norbury et al., 1997), thereby providing these cells with a robust mechanism of non-specific foreign antigen uptake.

## Methods to study macropinocytosis in DCs

Since foreign-antigen uptake and proteolysis are such important functions of DCs, a variety of tools have been developed to study the mechanisms of antigen uptake in these cells. Quantitating the uptake of fluorescently-labeled molecules [often BSA, dextran, or ovalbumin (OVA)] or the fluorescent dye Lucifer Yellow (LY) is a standard method for measuring antigen uptake via macropinocytosis. However, in many of the studies performed to date it has been difficult to unambiguously distinguish between macropinocytosis and receptor-mediated endocytosis as the mechanims of “antigen” uptake in DCs. Whereas both fluorescently-labeled dextran and OVA have been used to measure uptake via “macropincytosis,” partial suppression of uptake by pretreatment with the mannose receptor-agonist mannan demonstrates that dextran and OVA are also internalized by mannose receptor-mediated endocytosis (Sallusto et al., 1995; Burgdorf et al., 2006; Platt et al., 2010). Thus, the choice of solute marker examined, and exact experimental protocol followed, are the key to knowing precisely which endocytic pathway is actually being assayed.

In addition, a number of inhibitors have been used to “specifically” study macropinocytosis. Inhibitiors of the amiloride family (amiloride, EIPA, HOE-694, and DMA) inhibit Na^+^/H^+^ exchanger activity that promotes actin polymerization during macropinocytosis. Sanglifehrin A and rapamycin are immunophilin-binding agents that exert immunosuppressive effects on DC functions including macropinocytosis (Sallusto et al., 1995; Hackstein et al., 2002, 2007). However, other studies revealed that these compounds also inhibit receptor-mediated endocytosis (Hackstein et al., 2002, 2007; Sarkar et al., 2005; Koivusalo et al., 2010). The same issue occurs when using PI3-kinase inhibitors (wortmannin and LY294002) and cytochalasins as inhibitors of macropinocytosis. These inhibitors function by blocking actin polymerization and therefore they not only reduce macropinocytosis but also inhibit phagocytosis in DCs (Araki et al., 1996, 2003). Low doses (<3 μM) of the PKCδ inhibitor Rottlerin significantly reduce uptake of Lucifer Yellow, but not transferrin, in DCs, suggesting that this compound preferentially blocks macropinocytosis in this cell type (Sarkar et al., 2005). However, Rottlerin has been shown to inhibit dectin-1-mediated phagocytosis of zymosan in monocytes when used at a concentration of 5 μM (Elsori et al., 2011), calling into question the specificity of this compound as an inhibitor of macropinocytosis. All the above data suggest that it is very difficult to specifically study the importance of macropinocytosis *per se* in DC antigen presenting function. More selective and efficient inhibitors need to be identified for investigation of the functional significance of macropinocytosis in the future.

## Regulation of macropinocytosis in DCs

Most DCs exist *in vivo* in a quiescent “immature” state that can be converted to an activated “mature” state by exposure to a wide variety of stimulatory ligands. Whereas immature DCs have a very robust process of constitutive macropinocytosis, DCs lose this ability upon activation/maturation. The original observation of this phenomenon was made in DCs generated from human blood monocytes *in vitro* (Sallusto et al., 1995). These *in vitro*-cultured immature DCs can take up about 1–1.5 pL of extracellular fluid per hour, representing nearly the entire volume of cell itself. Addition of stimuli that induce the maturation of DCs (including TNF-α, IL-1β, CD40L, and a variety of TLR-ligands) profoundly suppresses macropinocytosis. Efficient macropinocytosis cannot be recovered when these stimuli are removed, demonstrating that an irreversible termination of macropinocytosis occurs upon DC maturation. Suppression of macropinocytosis upon DC maturation has been shown in mouse BMDC (Garrett et al., 2000) and in *in vitro* activated spleen DCs (West et al., 2000).

As in other non-immune cells, macropinocytosis in DCs is controlled by the Rho GTPase Cdc42 and Rac-dependent remodeling of the actin cytoskeleton. Immature DCs possess large amounts of GTP-bound Cdc42 whose expression is greatly diminished upon DC activation (Garrett et al., 2000). Injection of dominant-negative forms of Cdc42 or Rac blocked macropinocytosis in immature DC, whereas overexpression of constitutively-active forms of these same GTPases restored macropinocytosis in mature DCs (Garrett et al., 2000), revealing a direct role of these proteins in the modulation of macropinocytosis in DCs. Interestingly, kinetic studies of macropinocytosis in DCs showed that TLR stimulation of DCs transiently enhanced antigen macropinocytosis in the first few hours, a process that was then followed by dramatic suppression of macropinocytosis hours later (West et al., 2004). LPS and other TLR ligands transiently activated MAP kinase activity and stimulated actin polymerization and disassembly of actin-rich podosomes, thereby resulting in increased accumulation of fluorescent antigen-filled macropinosomes in DCs. However, this acute actin remodeling was not dependent on Rac or Cdc42 (West et al., 2004). It is thought that this transient burst in macropinocytosis in immature DCs serves to maximize antigen capture and presentation of relevant antigens associated with the inflammatory source of DC activation. It is interesting to note that TLR signaling promotes the formation of phagolysosomes following phagocytosis (Blander and Medzhitov, 2006), and it is an intriguing possibility that TLR signaling enhances not only the kinetics of antigen uptake following macropinocytosis but also enhances the “maturation” of macropinosomes into proteolytic antigen processing compartments.

The finding that macropinocytosis is dramatically suppressed upon DCs maturation fits well with the idea that DCs specifically acquire pathogens at the time of their activation *in vivo* and that *after* activation additional pathogen uptake is unnecessary (or even undesirable). It has therefore been widely accepted that immature DCs are the primary antigen samplers of peripheral tissues and that maturation convert DCs from efficient antigen *sampling* cells to efficient antigen *presenting* cells. There are, however, compelling data challenging this hypothesis. Drutman et al. showed that injection of mice with the inflammatory mediators LPS or CpG resulted in efficient *in vivo* DC maturation. Surprisingly, subsequent immunization of these “*in vivo* activated” mice with the model antigen OVA resulted in efficient stimulation of OVA-specific CD8^+^ and CD4^+^ T cells both *ex vivo* and *in vivo*, suggesting that *in vivo* activated DCs are still able to take up antigens and present them to T cells (Drutman and Trombetta, 2010). In contradiction to results obtained *in vitro*, direct measurements of antigen uptake confirmed that antigen delivered *in vivo* was internalized by macropinocytosis equally well by immature DCs and *in vivo* matured DCs. Importantly, in this study the authors confirmed the many previous findings that *in vitro* activation almost completely blocks macropinocytosis in DCs, highlighting the differences in the regulation of macropinocytosis in DCs activated *in vitro* versus DCs activated *in vivo*. Platt et al. also found that mature spleen DCs isolated from mice injected with LPS possessed robust macropinocytosis activity and were able to stimulate OVA-specific CD4^+^ T cells when assayed *ex vivo* (Platt et al., 2010), supporting the idea that *in vivo* activation of DCs does not profoundly reduce macropinocytosis and that *in vivo* activated DCs can generate pMHC-II capable of stimulating naïve CD4^+^ T cells.

The robust ability of *in vivo* matured DCs to internalize soluble antigen and subsequently generate pMHC-II complexes capable of stimulating antigen-specific T cell has not been seen in other studies. While Young et al. also found that spleen DCs from control mice and CpG-injected (i.e., *in vivo* matured)-mice internalized comparable amounts of soluble OVA antigen *ex vivo* (Young et al., 2007), in their study *in vivo*-activated spleen DCs were unable to stimulate OVA-specific CD4^+^ T cells (Young et al., 2007). The authors attributed this finding to impaired formation of MHC-II-peptide complexes in the mature DCs (due to the down-regulation of MHC-II biosynthesis) but not to impaired antigen uptake. Despite conflicting results regarding the extent of T cell activation by *in vivo*-activated DCs, each of these studies demonstrates that unlike *in vitro* matured DCs, *in vivo* matured spleen DCs can still internalize foreign soluble antigens, most likely via macropinocytosis. It is important to remember, however, that simply internalizing antigens is not sufficient for APC function; DCs must generate antigen-specific pMHC-II complexes from the internalized antigens in order to activate antigen-specific T cells *ex vivo* or *in vivo*. Factors that affect pMHC-II formation in immature vs. mature DCs [such as diminished biosynthesis of MHC-II (Cella et al., 1997), altered intracellular localization of MHC-II (Turley et al., 2000), or differences in endo/lysosomal proteinase activity (Trombetta et al., 2003)] will contribute to the efficiency of pMHC-II formation in mature DCs regardless of whether macropinocytosis is intact or not, as highlighted by the findings of the abovementioned studies.

There is also evidence showing that the extent of macropinocytosis varies among different types of DCs. Conventional DCs (cDCs) in the spleen capture significantly more soluble fluorescent OVA than plasmacytoid DCs (pDCs) do *in vitro*, however this difference was not seen *in vivo* (Young et al., 2008). While CD8^+^ and CD8^−^ cDCs internalize similar amounts of soluble OVA and BSA *in vitro*, these DC subsets target the internalized antigens into distinct intracellular compartments and present them to different classes of T cells (Schnorrer et al., 2006). In lung parenchyma, CD11b^hi^CD103^−^ DCs preferentially internalize soluble OVA protein for presentation on MHC-II to CD4^+^ T cells, whereas CD11b^low^CD103^+^ DCs preferentially phagocytose OVA-coated latex beads for presentation on MHC-I to induce CD8^+^ T cell responses (Jakubzick et al., 2008). Finally, in the skin Langerin^−^ dermal DCs internalize more soluble OVA than do Langerin^+^ epidermal DCs (Sparber et al., 2010). However, it is unclear whether OVA uptake in these distinct DC populations is mediated solely via macropinocytosis, as it has been shown that Langerin^−^ dermal DCs express higher amounts of OVA-binding mannose receptors than do Langerin^+^ epidermal DCs (Turville et al., 2002).

## Intracellular trafficking of pinocytosed antigens

Since one of the major functions of DCs is to process extracellular antigens into small peptides capable of generating pMHC-II complexes, the delivery of foreign antigens to the appropriate antigen-processing compartment is a crucial step in the induction of T cell responses. Sallusto et al. showed that immature DCs internalized and delivered the fluid phase marker FITC-dextran to a large compartment that contains MHC-II, cathepsin D, and LAMP-1, suggesting that macropinocytosed antigen was transported to late endosomes/lysosomes for degradation and pMHC-II formation (Sallusto et al., 1995). Subsequent studies confirmed this intracellular routing pathway of macropinocytosis. Macropinocytosed LY accumulates in late endosomes/lysosomes identified by Lysotracker but not in organelles expressing the early endosomal markers Rab5 and EEA-1 (Burgdorf et al., 2007). All these studies demonstrate that antigen endocytosed within macropinosomes is targeted to late endosomal/lysosomal MHC-II antigen processing compartments that are specialized for induction of CD4^+^ T cell responses.

Unlike macropinocytosis, receptor-mediated endocytosis can deliver antigens to distinct classes of endosomes in DCs. Mannose receptor-endocytosed OVA is preferentially delivered to an early endosomal compartment, and antigen targeting to this compartment generally leads to poor OVA processing and MHC-II presentation to CD4^+^ T cells (Burgdorf et al., 2007). By contrast, Fcγ receptor or DEC205-endocytosed OVA is transported rapidly to late endosomes/lysosomes for very efficient MHC-II antigen processing and presentation to OVA-specific T cells (Platt et al., 2010). These studies demonstrate that lysosomal targeting (and presumably proteolysis) of internalized antigens enhances MHC-II-restricted antigen presentation to CD4^+^ T cells.

## Significance of macropinocytosis for DC function

All APC subtypes are able to take up extracellular molecules, antigens, and nutrients. Macrophages primarily use surface receptor-dependent phagocytosis to engulf pathogens and apoptotic cells (Aderem and Underhill, 1999), while B cells primarily use their antigen-specific B cell receptor (BCR) to target specific antigens to antigen processing compartments (Lanzavecchia, 1990). By contrast, DCs use macropinocytosis as a major mechanism to sample their microenvironment. Thus efficient, constitutive macropinocytosis distinguishes DCs from macrophages and B cells as APCs capable of sampling tissues without regard to endocytic “receptor” specificity.

In the absence of infection or inflammation, immature DCs use macropinocytosis to constitutively capture self-antigen and present self-pMHC-II complexes to induce regulatory T cells in lymphoid organs (Steinman et al., 2003; Wilson et al., 2004), strongly suggesting that macropinocytosis contributes to DC-induced peripheral tolerance. Recent studies showed that immature peripheral plasmacytoid DCs (pDCs) can transport OVA peptide or OVA expressed in a tissue-specific manner to the thymus and induce clonal depletion of OVA-specific thymocytes (Bonasio et al., 2006; Hadeiba et al., 2012), indicating that constitutive macropinocytosis of antigens by DCs in the periphery may also contribute to the establishment of central tolerance. However, given the demonstration that some fraction of OVA enters cell by receptor-mediated endocytosis (Burgdorf et al., 2007), it remains an open question as to whether macropinocytosis alone is responsible for central tolerance induction in the thymus. Further studies are needed to determine whether, and to what extent, DC macropinocytic uptake of peripheral antigens contributes to central tolerance.

In the presence of infection or under inflammatory conditions, DCs employ macropinocytosis to internalize the soluble pathogenic antigens, degrade them into antigenic peptides, and present these antigenic pMHC-II complexes to CD4^+^ T cells. A number of studies have shown that macropinocytosis is important for MHC-II antigen presentation by DCs both *in vitro* and *in vivo*. Administration of macropinocytosis inhibitors not only reduces the amount of antigen taken up by DCs but also limits their capacity to stimulate antigen-specific CD4+ T cells. This finding has been validated using various model systems, including adoptive transfer of alloantigen-pulsed DCs into syngeneic mice as well as in *ex vivo* studies using antigen-pulsed APCs to stimulate a wide-variety of mouse and human antigen-specific CD4^+^ T cells (Sarkar et al., 2005; Hackstein et al., 2007; von Delwig et al., 2006; Drutman and Trombetta, 2010). Together, these studies demonstrate that interfering with antigen uptake via macropinocytosis results in impaired MHC-II antigen presentation by DCs.

Although macropinocytosis plays an important role in DC-mediated immune protection, pathogens can exploit the macropinocytosis machinery to gain access to host cells. Recent studies have shown that many different viruses use the macropinocytosis pathway to enter host cells and cause infection. Viruses possessing this ability include vaccinia virus, adenovirus 3, echovirus 1, coxsackievirus B, Herpes simplex virus 1, and human immunodeficiency virus (Mercer and Helenius, 2009). Each of these viruses activates small GTPases or kinases that stimulate macropinocytosis in host cells, thereby enhancing viral entry. These viruses colocalize with fluid-phase makers within infected cells, providing another line of evidence that viruses enter host cells through macropinocytosis. Although these studies were not directly performed using DCs as host cells, there is no reason to believe that these viruses do not also affect macropinocytiosis in DCs. One consequence of macropinocytosed vaccinia virus into DCs is that DC maturation itself is suppressed (Engelmayer et al., 1999), thereby reducing the possibility of an anti-vaccinia virus immune response. HIV-1 enters DCs by both receptor mediated endocytosis and macropinocytosis, however it is the macropinocytosis pathway that contributes to DC-mediated HIV-1 trans-infection of CD4^+^ cells (Wang et al., 2008), revealing a role of macropinocytosis in propagating HIV infection. These data therefore provide new insights into the role of macropinocytosis in DC-mediated immune responses.

## Conclusion

It is important to note that most of our current knowledge of the role of macropinocytosis in DC function has been acquired using *in vitro* culture systems. The contribution of macropinocytosis for DC function has not been fully tested *in vivo*, however this would require development of effective strategies to specifically disrupt macropinocytosis *in vivo*. Discovering specific inhibitors or generating mice containing mutations of known macropinocytosis regulators, such as Rac1 and Cdc42, is required to fully understand the contribution of this endocytic pathway to DC function in a physiological setting.

### Conflict of interest statement

The authors declare that the research was conducted in the absence of any commercial or financial relationships that could be construed as a potential conflict of interest.
